# Pharmacogenetic interventions to improve outcomes in patients with multimorbidity or prescribed polypharmacy: a systematic review

**DOI:** 10.1038/s41397-021-00260-6

**Published:** 2022-02-22

**Authors:** Joseph O’Shea, Mark Ledwidge, Joseph Gallagher, Catherine Keenan, Cristín Ryan

**Affiliations:** 1 School of Pharmacy and Pharmaceutical Sciences, Trinity College Dublin, Dublin, Ireland; 2 School of Medicine and Medical Science, University College Dublin, Dublin, Ireland; 3 School of Medicine, Trinity College Dublin, Dublin, Ireland

**Keywords:** Health services, Public health, Personalized medicine, Genetic testing, Pharmacogenomics

## Abstract

Conventional medicines optimisation interventions in people with multimorbidity and polypharmacy are complex and yet limited; a more holistic and integrated approach to healthcare delivery is required. Pharmacogenetics has potential as a component of medicines optimisation. Studies involving multi-medicine pharmacogenetics in adults with multimorbidity or polypharmacy, reporting on outcomes derived from relevant core outcome sets, were included in this systematic review. Narrative synthesis was undertaken to summarise the data; meta-analysis was inappropriate due to study heterogeneity. Fifteen studies of diverse design and variable quality were included. A small, randomised study involving pharmacist-led medicines optimisation, including pharmacogenetics, suggests this approach could have significant benefits for patients and health systems. However, due to study design heterogeneity and the quality of the included studies, it is difficult to draw generalisable conclusions. Further pragmatic, robust pharmacogenetics studies in diverse, real-world patient populations, are required to establish the benefit of multi-medicine pharmacogenetic screening on patient outcomes.

## Introduction

The population is rapidly ageing. By 2050, the number of elderly people (≥65 years) worldwide is expected to increase two-fold [1]. There is a well-recognised association between ageing and the presence of multimorbidity (two or more chronic conditions) and polypharmacy (the prescribing of four or more medications, although various definitions are used) [2–5]. In older persons, the prevalence of multimorbidity is estimated to range from 55% to 98% [6]. Furthermore, the occurrence of associated polypharmacy is increasing. A population database analysis in Scotland found that between 1995 and 2010, the proportion of adults dispensed five or more medicines doubled to 20.8% and the proportion of elderly patients prescribed ≥10 medicines more than tripled to 17.2% [7]. However, the issue of multimorbidity and polypharmacy is not restricted to age, with a substantial number of young and middle-aged people also affected [4, 7]. Several negative health outcomes are associated with multimorbidity and polypharmacy, including increased healthcare utilisation, mortality rates, healthcare costs, and poorer health-related quality of life [6, 8–11]. Nevertheless, the organisation and delivery of healthcare, as well as the development of clinical guidelines, are primarily built around singular diseases [4, 12].

A more holistic and integrated approach to healthcare delivery and medicines optimisation is needed to carefully identify the correct balance between appropriate and inappropriate polypharmacy for each multimorbid patient. The World Health Organisation (WHO) has emphasised the importance of refining healthcare systems to enable safer primary care for those with multimorbidity through personalisation of treatments and by combining best available evidence with clinical knowledge and judgement [13]. The National Institute for Health and Care Excellence (NICE) guidelines for multimorbidity management and the Scottish Government’s guidance on polypharmacy also advocate for a personalised approach to care [14, 15]. Such individualised approaches aim to improve treatment outcomes and appropriate polypharmacy by reducing inappropriate treatment burden and uncoordinated care, thereby avoiding medication-related problems, such as adverse drug events and drug interactions.

Drug interactions are associated with both appropriate treatment of chronic diseases and a majority of preventable drug-related hospitalisations (up to 87% in some studies) [7, 16]. For the purposes of this review, drug interactions are defined as drug-drug, drug-gene and drug-drug-gene interactions (those caused by a combination of drug-drug and drug-gene interactions) [17]. Drug-drug-gene interactions may involve inhibitory, induction or phenoconversion interactions, whereby the genetic variant and the perpetrator drug combine to act on transporter or metabolism pathways to significantly alter drug concentrations [17]. Pharmacogenetic analysis enables assessment of these gene-based variations in drug responses, which is significant as genetic polymorphisms are estimated to cause 15–30% of individual drug response variability [18], and >95% of all individuals carry at least one actionable genotype when tested for a panel of up to 12 genes [19–21]. Therefore, pharmacogenetic testing offers clinicians the opportunity to act prospectively rather than retrospectively, enabling the provision of the right medication at the right dose at the right time to individual patients [22]. Although the literature suggests that drug-gene and drug-drug-gene interactions are prevalent and clinically relevant [23–25], pharmacogenetics is not considered in the WHO, NICE and Scottish guidelines, and is rarely applied as part of medicines optimisation despite its enormous potential [13–15, 26]. Furthermore, two Cochrane reviews investigating interventions to improve outcomes for patients with multimorbidity and polypharmacy were found to have uncertain effectiveness [2, 3]; however, interventions incorporating pharmacogenetics were not identified.

Countless pharmacogenetic studies have been performed in recent years, yielding a substantial body of knowledge on gene-based variations affecting drug susceptibility. Evidence for the efficacy of pharmacogenetics to guide prescribing has been predominantly guided by studies and systematic reviews focused on single drug-gene or disease-gene pairs. For example, the various studies on pharmacogenetic-guided abacavir [27], anticoagulant [28–31], antidepressant [32–34], antipsychotic [35], clopidogrel [36], statin [37] and thiopurine therapy [38]. As a result, pharmacogenetics is gaining momentum in healthcare delivery in some countries, with various completed and ongoing implementation studies in the US, Canada, Europe, and Asia [39–42]. Worldwide, pharmacogenetic testing largely remains within the remit of specialist secondary and tertiary care settings [42, 43]. Outside of these environments, pharmacogenetics is emerging in primary care, where importantly, most prescribing and dispensing of medicines occurs [44]. Community pharmacy pharmacogenetic testing models have been investigated and demonstrate promise [45–47]. Furthermore, in the UK, the NHS England plan to incorporate pharmacogenetics in primary care by 2025 through adoption of a pre-emptive panel-based strategy for drug-gene pairs with the most evidence of (cost-)effectiveness [48].

Such clinical evidence is available in guidelines published by the Clinical Pharmacogenetics Implementation Consortium (CPIC) and the Dutch Pharmacogenetics Working Group (DPWG), providing actionable, genotype-based prescribing recommendations [49]. CPIC and DPWG have independently reviewed over 100 drug-gene interactions and have actionable recommendations for 60 and 55 individual drug-gene interactions respectively [50]. Despite established guidelines, the application of pharmacogenetics in routine patient care has been slow. Several barriers are frequently cited, including pharmacogenetics education, conflicting conclusions on clinical utility and cost-effectiveness, regulatory and reimbursement concerns, the need for informatics to support pharmacogenetics-informed prescribing decisions, and concerns over data sharing as well as other ethical, legal, and social implications surrounding pharmacogenetics [42, 43, 51–55]. It is envisaged that overcoming these barriers will provide the impetus for the widespread adoption of pharmacogenetic guidelines, enabling the realisation of the potential of pharmacogenetics [41].

Consequently, pharmacogenetics may have a role in improving the current approaches to medication usage, with potential for improving appropriate polypharmacy and preventing medication-related problems. However, the effectiveness of multi-gene, multi-drug, multi-disease pharmacogenetic interventions in adults with multimorbidity or prescribed polypharmacy is yet to be established. This systematic review aimed to establish the efficacy of such interventions in all healthcare settings, and to inform the implementation of pharmacogenetic-guided therapy into clinical practice.

## Results

### Search results

**Fig. 1:**
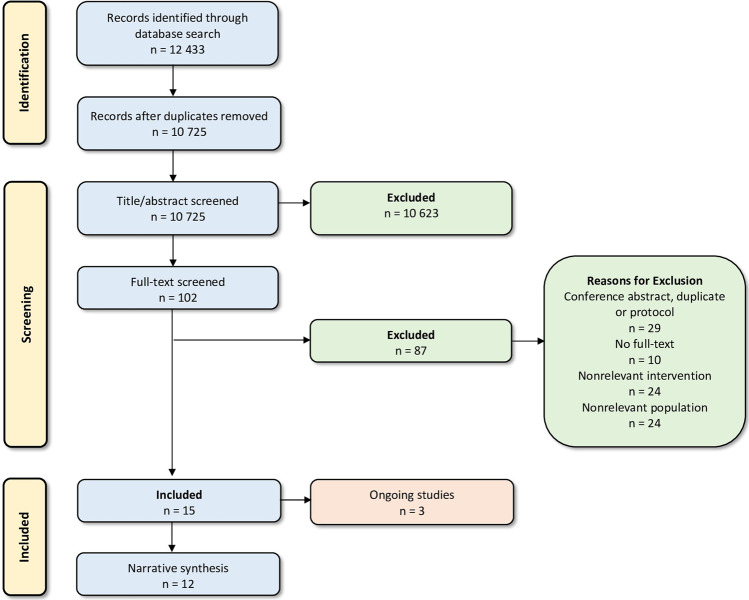
PRISMA flow diagram. Flow of information through the different phases of the present systematic review (number of records identified, excluded, and included). Excluded studies with reasons can be found in Supplementary Table 1.

Through the database searches, 12,433 records were retrieved, and 10,725 records were screened after de-duplication. Of these, 10,623 studies did not meet the inclusion criteria, and were excluded. Following assessment of the remaining 102 records in full text, 87 were excluded. Reasons for exclusion can be found in the PRISMA Flow Diagram (Fig. 1) and in the Supplementary Materials (Supplementary Table 1). Fifteen studies were eligible for inclusion, three of which are ongoing. Therefore, the narrative synthesis included twelve studies.

### Study characteristics

Twelve studies investigated multi-gene, multi-drug, multi-disease pharmacogenetic interventions in adults with multimorbidity and polypharmacy (Table 1). Six non-comparative studies [56–61], three observational studies [62–64], and three randomised controlled trials were included [65–67]. Meta-analysis was not suitable for the randomised studies; there was clinical and methodological diversity in these studies owing to variability in the outcomes assessed. The studies were predominantly US-based [56, 57, 59–61, 63–67], with the remainder conducted in Canada [58] and the Netherlands [62]. The comparative studies often used a control group untested for pharmacogenetics [64–66], or the control group was tested and the results withheld [67]. In the nested case-control and cross-sectional study, each participant underwent pharmacogenetic testing; in the former, comparisons were made between cases with frequent and controls with infrequent hospitalisations [63], while the latter made comparisons against a group lacking drug-gene interactions [62].

**Table 1:** Characteristics of included studies.

Source	Study design	Study description	Participants	Mean number of comorbidities	Mean number of medications
Randomised trials					
Elliott 2017 [66] United States	Randomised trial	IG: Pharmacist-led MTM on patients undergoing PGx testing followed by development of DDI, DGI and DDGI risk profiles using YouScript CDST. PGx test results and prescribing suggestions forwarded to physicians. CG: Comparisons made against an untested group who received usual care (standard pharmacist MTM). Genes: CYP2C9, CYP2C19, CYP2D6, CYP3A4, CYP3A5 and VKORC1.	Elderly polypharmacy patients IG = 57 CG = 53	Not reported	IG = 11.6 CG = 11.8
Kim 2018 [65] United States	Randomised trial (post-hoc analysis)	IG: Pharmacist-led MTM using YouScript with and without PGx (IG1 and IG2 respectively). IG1 underwent PGx testing followed by development of DDI, DGI and DDGI risk profiles. PGx test results and prescribing suggestions forwarded to their physicians. IG2 (untested for PGx) was used to assess effect of CDST alone. CG: Comparisons made against an untested group who received usual care (standard pharmacist MTM). Genes: CYP2C9, CYP2C19, CYP2D6, CYP3A4, CYP3A5 and VKORC1.	Polypharmacy patients IG1 = 58 IG2 = 180 CG = 104	IG1 = 6.5 ± 2.8 IG2 = 6.6 ± 2.6 CG = 6.2 ± 2.2	IG1 = 11.5 ± 4.1 IG2 = 11.5 ± 4.3 CG = 11.2 ± 3.8
Saldivar 2016 [67] United States	Randomised trial (non-comparative results)	All patients tested; those with passing results randomised to IG or CG. IG: Pharmacist-led MTM using IDgenetix to generate DDI and DGI recommendations. PGx test results and prescribing suggestions forwarded to their physicians. Results listed only for this group (*n* = 132). CG: PGx results withheld. Genes: CYP2C9, CYP2C19, CYP2D6, CYP3A4, CYP3A5, VKORC1, CYP1A2, HTR2A, HTR2C, SLC6A4, SLC6A2, COMT, OPRM1, SLCO1B1, MTHFR, F2 and F5.	Patients in a long-term care facility *n* = 132	Not reported	12.0
Non-randomised trials (observational and non-comparative studies)					
Brixner 2016 [64] United States	Non-concurrent cohort study	IG: Pharmacist-led MTM on patients undergoing PGx testing followed by development of DDI, DGI and DDGI risk profiles using YouScript CDST. PGx test results and prescribing suggestions forwarded to their physicians. CG: Comparisons made against an untested historical cohort (matched on key variables via a propensity score method). Genes: CYP2C9, CYP2C19, CYP2D6, CYP3A4, CYP3A5 and VKORC1.	Elderly polypharmacy patients IG = 205 CG = 820	Not reported	4.0^a^
Finkelstein 2016 [59] United States	Non-comparative case series study	Participants offered PGx testing by their treating physician to optimise their therapy. GENETWORx was used for analysis. The testing facility provided detailed findings reports and basic education materials explaining the general principles of PGx testing. Genes: CYP2C9, CYP2C19, CYP2D6, CYP3A4, CYP3A5 and VKORC1.	Elderly polypharmacy patients *n* = 3	7.0	20.3
Finkelstein 2016 [63] United States	Nested case-control study	Cases: chosen from eligible patients with high rates of hospitalisations. Controls: included eligible patients with infrequent hospitalisations matched to cases on age, race, ethnicity and chronic disease score. PGx testing performed on all patients. GENETWORx used for the analysis. The testing facility provided PGx reports and education materials explaining the general principles of PGx testing. DGI severity was confirmed by an independent pharmacist review. Genes: CYP2C9, CYP2C19, CYP2D6, CYP3A4, CYP3A5 and VKORC1.	Elderly polypharmacy patients IG = 6 CG = 6	IG = 8.2 ± 1.2 CG = 8.2 ± 2.0	IG = 14.3 ± 5.3 CG = 14.0 ± 2.9
Keine 2019 [60] United States	Non-comparative case series study	Patients with a family history of Alzheimer’s disease, mild cognitive decline or mild Alzheimer’s disease were enroled. uMethod Health’s precision medicine platform was used to analyse DDIs DGIs, anticholinergic burden and depression-inducing drugs. PGx prescribing suggestions reviewed by a physician and forwarded to patients. Genes: Gene panel is not detailed.	Elderly polypharmacy patients *n* = 295	Not reported	11.5
Lee 2019 [61] United States	Non-comparative case series study	Genotyped 1200 Patients Project participants analysed for hospitalisations (*n* = 20) to examine medication changes, actionable PGx information and potential prescribing actions using CPIC, FDA and Genomic Prescribing System CDST PGx information. Genes: CYP2C9, CYP2C19, CYP2D6, CYP4F2, VKORC1, SLCO1B1, KIF6, GNB3, LTC4S, ADD1 and GLCCI1.	Polypharmacy outpatients *n* = 867	7.6	8.9
Papastergiou 2017 [58] Canada	Non-comparative case series study	Pharmacists trained in PGx enroled patients they thought would benefit from the service. Geneyouin provided the tests and evidence-based reports (CPIC and FDA) highlighting patients’ metabolic profiles and risk medications. PGx test results and prescribing suggestions forwarded to physicians. Genes: CYP2C9, CYP2C19, CYP2D6, CYP3A4, CYP3A5, VKORC1, CYP1A2, OPRM1 and SLCO1B1.	Community pharmacy patients *n* = 100	Not reported	4.9
Reynolds 2017 [56] United States	Non-comparative case series study	Physicians ordered PGx testing for eligible patients; genotypes were correlated to predicted phenotypes on the PRIMER report. Pharmacists performed MTM (DDIs and DGIs) and ranked the severity of interactions. PGx test results and prescribing suggestions forwarded to physicians. Genes: CYP2C9, CYP2C19, CYP2D6, CYP3A4, CYP3A5, VKORC1, CYP1A2, SLC6A4, COMT, OPRM1, SLCO1B1, F2, F5 and MTHFR.	Polypharmacy patients *n* = 705	Not reported	12.0
Sugarman 2016 [57] United States	Non-comparative case series study	Pharmacist-led MTM using IDgenetix to generate DDI and DGI recommendations. PGx test results and prescribing suggestions forwarded to their physicians. Genes: CYP2C9, CYP2C19, CYP2D6, CYP3A4, CYP3A5, VKORC1, CYP1A2, HTR2A, HTR2C, SLC6A4, SLC6A2, COMT, OPRM1, SLCO1B1, and MTHFR.	Patients in a long-term care facility *n* = 112	Not reported	19.0
Van der Wouden 2019 [62] The Netherlands	Cross-sectional study	Pharmacists requested PGx tests for eligible patients to guide therapy. DPWG guidelines provided the recommendations that were sent to pharmacists and patients’ physician. PGx data was saved in both electronic medical records for future use; follow-up was 2.5 years. Patients put into three groups: [1] did not encounter a DGI or encountered a DGI and healthcare professional [2] adhered or [3] did not adhere to guidelines. Genes: CYP2C9, CYP2C19, CYP2D6, CYP3A5, SLCO1B1, TPMT, VKORC1 and DPYD.	Community pharmacy patients G1 = 138 G2 = 49 G3 = 9	G1 = 4.4 ± 2.4 G2 = 4.9 ± 2.6 G3 = 4.4 ± 2.3	G1 = 3.9 ± 3.4 G2 = 4.0 ± 2.9 G3 = 4.4 ± 3.0

*ADD* alpha-adducin, *CDST* clinical decision support tool, *CG* control group, *COMT* catechol-O-methyltransferase, *CPIC* Clinical Pharmacogenetics Implementation Consortium, *CYP* cytochrome P450, *DPWG* Dutch Pharmacogenetic Working Group, *DPYD* dihydropyrimidine dehydrogenase, *DDI* drug-drug interaction, *DDGI* drug-drug-gene interaction, *DGI* drug-gene interaction, *ED* emergency department, *F2* Factor II prothrombin, *F5* Factor V Leiden, *FDA*, U.S. Food and Drug Administration, *G* group, *GLCCI* glucocorticoid induced, *GNB* G protein subunit beta, *HTR* 5-hydroxytryptamine receptor, *IG* intervention group, *KIF* kinesin family member, *LTC4S* leukotriene C4 synthase, *MTHFR* methylenetetrahydrofolate reductase, *MTM* medication therapy management, *PGx* pharmacogenetic, *OASIS* Outcome and Assessment Information Set, *OPRM* opioid receptor mu, *SLC* solute carrier (serotonin transporter), *SLCO* solute carrier organic anion transporter, *TPMT* thiopurine methyltransferase, *VKORC* vitamin K epoxide reductase complex.

^a^D. Brixner contacted; estimated the majority were on four or more medications.

The included studies predominantly took place in primary care, except for one study assessing the impact of pharmacogenetic profiling on in-hospital prescribing [61]. The majority of the included studies involved explicit pharmacist-led medication management with recommendations forwarded to patients’ physicians. Mean age ranged from 57 to 78 years, the proportion of males ranged from 31% to 67%, the mean number of conditions and medications ranged from 5 to 8 and 4 to 20, respectively, and most participants were of Caucasian ethnicity (67–99%). Similarities were evident in the genetic testing approach, with a core panel consisting of CYP2C9, CYP2C19, CYP2D6, CYP3A4, CYP3A5 and VKORC1. Various clinical decision support (CDS) systems were employed: YouScript [64–66], GENETWORx [59, 63], IDgenetix [57, 67], GeneYouIn [58], uMETHOD Health [60], Genomic Prescribing System [61], and PRIMER [56].

### Summary of results

Four studies investigated the impact of pharmacogenetic interventions on healthcare utilisation. Reductions in hospitalisations and emergency department visits were observed following genetic testing and medicines optimisation [64, 66]. Brixner et al. reported reductions in hospitalisations and emergency department visits by 40% (*p* < 0.05) and 70% (*p* < 0.001), respectively [64], while Elliott et al. found reductions of 52% (*p* < 0.01) and 42% (*p* < 0.05), respectively [66]. A 47% increase in outpatients visits (*p* < 0.0001) in patients undergoing pharmacogenetic testing was reported by Brixner et al. [64]. Elevated rates of hospitalisation in elderly patients with pharmacogenetic polymorphisms was recorded by Finkelstein et al. (*p* < 0.05) [63]. Van der Wouden et al. found no significant differences in healthcare utilisation [62].

Estimated improvements in healthcare costs were reported in several studies. Brixner et al. found the cost of genetic testing was nearly or completely offset by savings resulting from decreased healthcare utilisation using mean and median national data. Using the mean, savings of $1132 per patient were made, while the median resulted in savings of $788 during the 16-week follow-up [64]. Elliott et al. modelled cost saving based on Medicare average all-cause readmission and emergency department cost, yielding savings of $4 382 per patient over the 8-week follow-up [66]. Two studies performed in long-term care facilities estimated cost savings of ~$1430 and $3000 over 2.3 years (average length of stay) [57, 67].

Actionable pharmacogenetic polymorphisms were ubiquitous. The potential for enhanced medication safety through drug interaction management was demonstrated, with up to seven gene-based drug interaction recommendations per patient [56–67]. Elliott et al. reported most patients carried at least one aberrant CYP variant [66]. Reynolds et al. identified drug-gene interactions in 78% of their participants [56]. Sugarman et al. reported medication change reasons were exclusively genetic in 28% of patients. For patients whose medications remained unchanged, a high proportion of genetic variations that could impact future prescriptions was observed [57]. Van der Wouden et al. identified targets for pharmacogenetic testing, reporting the number of newly initiated prescriptions with potential drug-gene interactions increases with age and number of comorbidities and comedications, but this was not found to be statistically significant [62]. Lee et al. suggested inpatient prescribing could be informed by pre-emptive genotyping those at risk of hospitalisation (elderly polypharmacy patients), as many prescriptions initiated in hospital included pharmacogenetic medications [61].

Clinical decision-making appeared reinforced by the interventions. Physicians followed between 30% and 79% of the medication-related problem recommendations [56, 58, 64–66]. Associations were found between physician acceptance and recommendation seriousness [64, 65] and if the recommendation involved pharmacogenetics [58]. Van der Wouden et al. demonstrated the importance of accurate record keeping to maintain the value of genetic testing. Within 2.5 years, a mean of 2.71 drugs for which results were available were prescribed; 24% of these were actionable drug-gene interactions. Pharmacists were found to be better able to record pharmacogenetic data than general practitioners (96% vs. 68%) [62]. Clinical outcomes, while prioritised in the core outcome sets [68, 69], were only described in Elliott et al. [66]. A statistically insignificant reduction in mortality was reported; however, the study was not powered for mortality and included this outcome post-hoc. Select quality metrics (such as pain, depression, and anxiety) and the number of falls were also recorded, demonstrating relatively small differences [66].

### Risk of bias

**Fig. 2:**
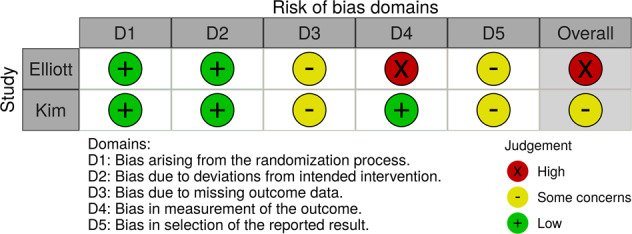
Risk of bias (RoB 2) plot of the domain-level judgements for randomised studies (65, 66). Risk of bias for randomised studies arising from the study design, conduct, and reporting, reported as ‘Low’ (green), ‘Some concerns’ (yellow) or ‘High’ (red) risk of bias.

**Fig. 3:**
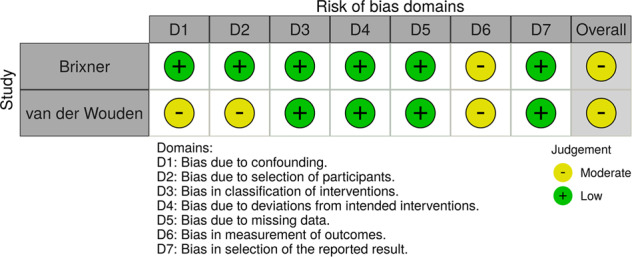
Risk of bias (ROBINS-I) plot of the domain-level judgements for non-randomised studies (62, 64). Risk of bias for non-randomised studies arising from the study design, conduct, and reporting, reported as ‘Low’ (green), ‘Moderate’ (yellow) or ‘High’ (red) risk of bias.

The risk of bias assessment is detailed in the Supplementary Materials (Supplementary Table 2) and summarised in Figs. 2, 3. Overall, the risk of bias ranged from moderate/some concerns to high risk. RoB 2 was used for two randomised studies [65, 66]. Elliott et al. was found to be at high risk of bias [66], while Kim et al. had some concerns [65]. ROBINS-I was used for two non-randomised studies [62, 64]; both were found to be at moderate risk of bias. The study by Saldivar et al. was designed as a randomised trial; however, it is an “initial assessment” and provides non-comparative results [67]. Similarly, the case-control study by Finkelstein et al. did not compare the effect of the intervention received [63]. It was not possible to assess non‐comparative studies because it is a prerequisite in ROBINS-I that there is a comparative study. Non‐comparative studies were considered at critical risk of bias mostly due to confounding factors [70].

### Summary of relevant ongoing studies

Three ongoing trials were eligible [41, 71, 72]. Recruitment is ongoing for Stingl et al. (DRKS00006256) and Delate et al. (NCT04120480), while the third trial by van der Wouden et al. (NCT03093818) is active but not recruiting. The German iDrug primary care randomised controlled trial (Stingl et al.) involves elderly multimorbid and polypharmacy patients randomised to receive an individual risk assessment (including drug-drug interactions and pharmacogenetics) or a standardised risk assessment (without individualised information) to analyse the effect this information has on adverse events [71]. Outcomes include mortality, healthcare utilisation, costs, medication changes, adverse drug reactions and quality of life. The estimated completion date is not reported.

In the United States, Delate et al. are conducting a pharmacist-led randomised controlled trial involving high-risk polypharmacy patients randomised to receive pharmacogenetic-guided treatment or usual care to determine clinical and economic effectiveness [72]. The investigators hypothesise that pharmacogenetic testing and pharmacist review of medication appropriateness will lower one-year healthcare utilisation and costs compared to controls. Outcomes include healthcare utilisation, costs, medication changes, medication congruence and adherence. The estimated completion date is December 2022.

The PREPARE randomised controlled trial (van der Wouden et al.) conducted in several European countries has an estimated completion date of April 2021, with results yet to be published [41]. In this trial, adults receiving a first prescription for one or more of 42 medications with a DPWG guideline were randomised to receive pharmacogenetic-guided treatment or usual care. In the intervention group, pharmacogenetic results may be used to guide medication and dose selection per DPWG guidelines. Patients receive a “Safety-Code card” containing their pharmacogenetic results, which can be used by other healthcare professionals during subsequent prescriptions. Outcomes include costs, adverse drug reactions, medication changes, quality of life, attitude towards and knowledge of pharmacogenetics, and physician and pharmacist adherence to DPWG guidelines.

### General process model

**Fig. 4:**
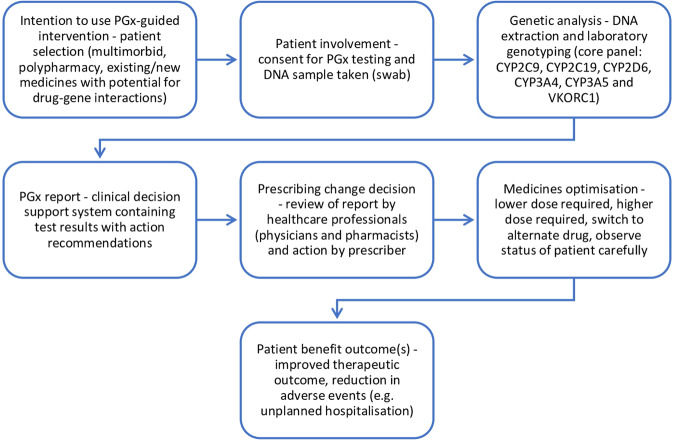
General process model for pharmacogenetic (PGx) interventions. Derived from the steps described in each of the studies, this PGx general process model outlines the steps required for a PGx intervention that can prompt medication changes, patient benefit, and reduce adverse events such as unplanned hospitalisation.

To aid the development of future pharmacogenetic interventions, a process diagram derived from the steps described in each of the included studies was produced (Fig. 4). This model outlines the steps required for a pharmacogenetic intervention that can prompt medicines optimisation, patient benefit and reduction in adverse events.

## Discussion

This is the first systematic review to examine the effectiveness of multi-drug pharmacogenetic interventions in the management of those with multimorbidity and/or prescribed polypharmacy. This study demonstrates that once the scope of the review extends beyond single drug-gene interactions there is limited available evidence. Following retrieval of 10,725 records, fifteen studies were eligible for inclusion, three of which are ongoing, limiting the conclusions that can be drawn. Nevertheless, the included study by Elliott et al. provides randomised controlled trial evidence, albeit in a small, selected population, in favour of the incorporation of pharmacogenetic testing in primary care to improve outcomes for those with multimorbidity and polypharmacy [66]. Interpretations from the other randomised studies are limited by the post-hoc design of Kim et al. and the lack of information provided by Saldivar et al. [65, 67]. In general, medicines optimisation approaches currently do not incorporate pharmacogenetics as a cause of medication-related problems [73, 74]. Conversely, pharmacogenetics is a source of such problems, and could add an important new dimension to conventional drug interaction assessment processes. Most of the search results were published in just the past decade, highlighting that pharmacogenetics is an emerging field. Three ongoing trials, one of which is being conducted throughout Europe (the PREPARE study), has recruited almost 7000 participants and will provide important new evidence [41]. These and more pragmatic studies in diverse, real-world patient populations, are required to establish the benefit of multi-medicine pharmacogenetics.

The heterogeneity of study designs employed in this space must be addressed. Lack of evidence from gold standard randomised controlled trials is frequently cited as a reason to delay pharmacogenetics implementation, despite a substantial evidence base and published guidelines. This necessity has been challenged [75–78]; many argue that the perceived mandatory requirement for prospective evidence to support the clinical validity of a pharmacogenetic test, prior to its implementation into routine care, is inappropriate and unreasonable [75–78]. There are suggestions to use alternative forms of evidence such as smaller-scale, non-randomised clinical studies, and strong observational evidence [52, 79–82]. However, limitations exist, and designs such as case-control and cohort studies, that are very common in pharmacogenetics, can be prone to confounding and various biases [79, 83]. Thus, the conclusions that can be drawn from included studies using such alternative designs are limited [56–64, 67]. This underlines the importance of and need for more robust, well-designed, pragmatic, randomised controlled trials with large sample sizes, such as the PREPARE study [41]. Further good quality evidence will help establish the true efficacy and utility of pharmacogenetics in personalised patient care, and to advance the discovery and development of appropriate patient outcome improvement strategies.

This systematic review provides various considerations for future studies. A collaborative, primary care approach involving physicians, pharmacists, and patients was shown to underly the implementation of pharmacogenetics (Fig. 4). With their training, frequent patient contact and role in medicines optimisation as well as medication surveillance, pharmacists may be leading candidates to manage incorporating pharmacogenetics into medicines optimisation [84]. Pharmacists’ knowledge of pharmacokinetics and pharmacodynamics may be useful in the clinical application of pharmacogenetics [85]. This is supported by the majority of studies included in this review involving pharmacist-led medication management, as well as by other pilot studies performed in pharmacy settings [45–47, 86, 87]. Studies have demonstrated pharmacists’ interest in incorporating pharmacogenetic services into their practices, albeit further education may be required [88–90]. Comprehensive continuing professional development courses, that developed in the PRIME study [39], may improve knowledge, readiness and comfort in applying pharmacogenetics to patient care [39, 91]. Both community pharmacists and general practitioners may have long-term, regular roles in the care of polypharmacy patients and are able to record the results of pharmacogenetics tests in their EHRs [62], a pivotal facilitator of clinical utility and cost-effectiveness of pharmacogenetic testing. Thus, pharmacogenetic testing in pharmacy practice may be considered appropriate and could play an important role in moving pharmacogenetics from research to patient care.

However, pharmacogenetics implementation barriers exist. Evidence of cost-effectiveness and testing utility is another major barrier to the broader implementation of pharmacogenetics [18, 42, 51, 53, 55]. There has been considerable debate around the optimal approach to pharmacogenetic testing in clinical practice, particularly the methodology of genotyping [92]. Some support reactive genotyping for a single drug-gene interaction at the point of prescribing, and others a pre-emptive panel-based approach prior to prescribing [92]. The former approach has several disadvantages. For instance, if patients receive prescriptions for multiple drugs with pharmacogenetic implications, they may require additional testing for multiple single genes. Here, the cost of testing is amplified, and treatment may be delayed awaiting the test results [62, 92]. Since the overall cost of panel-based and single gene tests are similar, a pre-emptive panel approach may alleviate these concerns, as highlighted by the PREDICT study [24]. The ordering of 14,656 genetic tests was avoided when data on multiple genes was available beforehand, thereby saving genotyping test costs by reducing the number of single tests by 60% [24]. The multi-drug, multi-gene scope of this review provided auspicious estimates of cost-savings [57, 64, 66, 67]. Considering the follow-up durations were short and that the pharmacogenetic test results are lifelong, the value obtained from a one-time expense of testing is likely to increase over time with ongoing patient management. Compared to the previous testing methods, whole genome sequencing offers more in-depth information [44]; however, the associated expense, immense data, extended test turnaround times, and complex interpretation arguably makes a panel-based approach a better suited technology for larger scale implementation at present [42, 44]. Another important consideration is the cost to the healthcare professional, for instance, unreimbursed time spent counselling, ordering pharmacogenetic tests, and conducting medicines optimisation [55].

Furthermore, primary care workforce education and support regarding pharmacogenetics and a proper infrastructure for the integration of pharmacogenetics are crucial to pave the way for accessible pharmacogenetics [41, 42, 51–55]. The latter barrier may be overcome by greater integration of pharmacogenetic results into electronic health records (EHRs), and development and deployment of CDS as part of EHRs [93]. In the US, several implementation studies integrating pharmacogenetic test results into the EHR and CDS systems have been initiated, such as the eMERGE-PGx, IGNITE, INGENIOUS, and PG4KDS studies [18, 41]. For healthcare systems with limited EHR infrastructure, the “Safety Code card” used in European PREPARE study may be a viable option to make pharmacogenetic data and CDS available [41, 94]. This card is part of a mobile-based CDS system that is independent of existing information technology infrastructures, and after scanning the quick response code, enables retrieval of patient-relevant pharmacogenetic dosing guidelines [94]. In lieu of a nationwide EHR, an approach such as this may ameliorate accessibility and sharing of pharmacogenetic results within and between different healthcare setting and healthcare professionals.

This review is strengthened by its pragmatic focus, examining the effectiveness of multi-drug pharmacogenetic-guided therapy in the care of those with multimorbidity or polypharmacy compared with the established literature focusing on one singular drug, disease, or limited drug-gene combinations. This review placed no restriction on language or geographical region, enabling a broader view of pharmacogenetic interventions internationally. Our review is also strengthened by the robust methodology used and was developed in accordance with the PRISMA statement and Cochrane tools were used to assess the risk of bias.

## Limitations

This study has some limitations to consider. First, only a narrative analysis was performed due to significant heterogeneity in the articles included. Thus, the results only provide a high-level representation of the impact of pharmacogenetic testing in patients with multimorbidity and/or polypharmacy. Second, we are constrained by the sparsity of evidence available on the efficacy of pharmacogenetics in this area. The dearth of gold-standard randomised controlled trial evidence in this area necessitated the need to include observational studies and non-comparative studies. Finally, studies undertaken in multimorbid populations with a specific focus on a single drug or drug class were not included, and we await the results of the largest, pragmatic study of pharmacogenetics undertaken to date [41].

## Conclusions

The incorporation of pharmacogenetic testing into the medicines optimisation process could have significant benefits for healthcare providers and for patients by reducing healthcare utilisation and costs, enhancing identification of clinically significant drug interactions, and improving clinical decision-making. Due to a lack of methodologically robust, high quality studies, small sample sizes, and relatively short follow-up durations, we found limited evidence on the efficacy of pharmacogenetic interventions to improve outcomes in patients with multimorbidity or prescribed polypharmacy. In one small, randomised study, encouraging results using a multi-gene, multi-disease, multi-drug pharmacogenetic approach as part of medicines optimisation by a pharmacist were found. We conclude that pharmacogenetic-guided therapy holds promise for individualising therapy; however, further robust, pragmatic studies, in all patient care settings, are required to establish the impact pharmacogenetic screening has on patient outcomes.

## Materials and methods

This systematic review was registered with PROSPERO (registration number CRD42020178126), and was developed in accordance with the PRISMA 2020 statement [95, 96]. Study inclusion was based on the Cochrane EPOC Checklist (which included randomised trials, non-randomised trials, controlled before-after studies, and interrupted time series analyses) [97], as well as observational and non-comparative studies. Broad study designs were included to ensure a comprehensive report on the available literature was produced.

### Search strategy

We systematically searched PubMed, Embase, Cochrane CENTRAL, CINAHL, AMED, and PsycInfo from inception to April 2020, using keywords and controlled vocabulary related to ‘pharmacogenetics’, ‘pharmacogenomics’, ‘multimorbidity’ and ‘polypharmacy’ without restricting the language of publication. The search strategy was developed in collaboration with an expert subject librarian. We conducted searches for ongoing or unpublished trials on ClinicalTrials.gov (www.clinicaltrials.gov), the EU Clinical Trials Register (www.clinicaltrialsregister.eu), and the WHO International Clinical Trials Register (http://apps.who.int/trialsearch). The search strategy is reported in the Supplementary Materials (Supplementary Table 3).

### Study selection

Studies were included if they met the following criteria: investigated interventions involving multi-gene, multi-drug, multi-disease pharmacogenetic-guided treatment; recruited participants ≥18 years experiencing multimorbidity (the presence of two or more chronic conditions in the same individual [2]) or prescribed polypharmacy (the prescribing of four or more medicines [3]); and reported on at least two outcomes derived from multimorbidity and polypharmacy consensus-based core outcome sets in Table 2 [68, 69]. We included all healthcare settings and interventions provided by any healthcare professional. Studies involving single gene, single drug, single disease pharmacogenetic interventions, malignancy, palliative care, human immunodeficiency virus and hepatitis were excluded. Study selection involved a two-phase screening and eligibility determination process by two reviewers independently through Covidence [98]. Initially, titles and abstracts were assessed for relevancy, followed by a full-text review.

**Table 2:** Combination of the core outcome sets for multimorbidity and polypharmacy.

**Clinical outcomes**	**Health systems-related outcomes**	**Patient knowledge and behaviour**	**Medication-related outcomes**	**Patient-related outcomes**	**Consultation-related outcomes**
Mortality^a^ Mental health^a^	Health service utilisation/ Hospitalisation Health care costs Quality of health care (patient-rated)	Patients’ knowledge Self-rated health Self-management behaviour Self-efficacy	Medication appropriateness^a^ Serious adverse drug reactions^a^ Medication regimen complexity^a^ Medication side effects^a^ Adherence Clinically significant drug interactions The number of ‘regular’ medicines prescribed Therapeutic duplication Prescribing errors	Quality of life/Health-related quality of life^a^ Falls^a^ Treatment/Medication burden Cognitive function Physical function Activities of daily living function Physical activity	Communication Shared decision making Prioritisation

^a^priority outcomes - all studies should consider them and then consider the others depending on the individual study.

### Data extraction

Data extraction was conducted by two reviewers independently, focusing on four domains: [1] characteristics of study (study design, sample size, follow-up duration, inclusion criteria), [2] patient demographics (age, gender, ethnicity, number of chronic conditions and concomitant medications), [3] details about the intervention (pharmacogenetic component(s), description of the intervention and control) and [4] study findings (outcomes and results). Additional information related to the publication (funding and conflicts of interest) was also collected to assess study quality.

### Quality assessment

The risk of bias was assessed by two reviewers independently using the Cochrane RoB 2 and ROBINS-I tools for randomised and non-randomised studies, respectively [70, 99]. The RoB 2 tool provides a framework for assessing the risk of bias against five domains: the randomisation process; deviations from the intended interventions; missing outcome data; measurement of the outcome; and selection of the reported result. ROBINS-I has seven domains: confounding; selection of participants into the study; classification of interventions; deviations from intended interventions; missing data; measurement of outcomes; and selection of the reported result. The potential sources of bias in RoB 2 were graded ‘low’, ‘some concerns’ or ‘high’, and ROBINS-I as ‘low’, ‘moderate’, ‘serious’ or ‘critical’. The risk of bias figures were generated using robvis [100].

### Presentation of results

Because of the heterogeneity in the methods and outcomes of included studies, it was not possible to conduct a meta-analysis. The findings of this study are reported as a narrative synthesis and include a description of the study characteristics and a summary of the study results.

## Supplementary information

Supplementary materials
