# Correction to: Pharmacogenetic interventions to improve outcomes in patients with multimorbidity or prescribed polypharmacy: a systematic review

**DOI:** 10.1038/s41397-022-00273-9

**Published:** 2022-03-30

**Authors:** Joseph O’Shea, Mark Ledwidge, Joseph Gallagher, Catherine Keenan, Cristín Ryan

**Affiliations:** 1 School of Pharmacy and Pharmaceutical Sciences, Trinity College Dublin, Dublin, Ireland; 2 School of Medicine and Medical Science, University College Dublin, Dublin, Ireland; 3 School of Medicine, Trinity College Dublin, Dublin, Ireland

**Keywords:** Health services, Public health, Personalized medicine, Genetic testing, Pharmacogenomics

Correction to: *Pharmacogenomics J* 10.1038/s41397-021-00260-6, published online 22 February 2022

In the original article, the legends to Figures 1, 2, 3 and 4 were incorrect. The original article has been corrected.

