# Supplementary material for: Pharmacogenetic interventions to improve outcomes in patients with multimorbidity or prescribed polypharmacy: a systematic review
Source: Pharmacogenomics J. 2022 Feb 22;22(2):89–99. doi: 10.1038/s41397-021-00260-6 (PMC8975737; doi:10.1038/s41397-021-00260-6)
Supplement: Supplementary file 1 — Supplementary materials [file 41397_2021_260_MOESM1_ESM.docx]

Pharmacogenetic interventions to improve outcomes in patients with multimorbidity or prescribed polypharmacy: a systematic review

SUPPLEMENTARY MATERIALS

# Supplementary Table 1: Characteristics of excluded studies

| **Study** | **Reason for exclusion** |
| --- | --- |
| Al-Zubiedi 2009 (1) | Conference abstract: full-text study retrieved ((2)) |
| Altar 2015 (3) | Wrong patient population: no evidence of multimorbidity or polypharmacy |
| Anderson 2007 (4) | Wrong patient population: no evidence of multimorbidity or polypharmacy |
| Anderson 2010 (5) | Wrong patient population: no evidence of multimorbidity or polypharmacy |
| Barley 2014 (6) | Wrong intervention: intervention lacked a pharmacogenetics component |
| Bielinski 2014 (7) | Protocol: full-text study retrieved ((8)) |
| Bielinski 2014 (9) | Duplicate: duplicate protocol for a retrieved full-text study ((8)) |
| Biskupiak 2015 (10) | Conference abstract: full-text study retrieved ((11)) |
| Biskupiak 2015 (12) | Duplicate: duplicate conference abstract for a retrieved full-text study ((11)) |
| Boels 2017 (13) | Wrong intervention: intervention lacked a pharmacogenetics component |
| Bradley 2018 (14) | Wrong patient population: no evidence of multimorbidity or polypharmacy |
| Brown 2017 (15) | Wrong patient population: no evidence of multimorbidity or polypharmacy |
| Brown 2020 (16) | Conference abstract: full-text study retrieved ((17)) |
| Burnette 2012 (18) | Wrong intervention: no intervention |
| Cervantes 2019 (19) | Conference abstract: full-text study unobtainable |
| Charland 2012 (20) | Conference abstract: full-text study retrieved ((21)) |
| Charland 2014 (21) | Wrong intervention: intervention involved a single drug-gene interaction |
| Chenoweth 2014 (22) | Wrong intervention: intervention lacked a pharmacogenetics component |
| Clarke 2011 (23) | Wrong intervention: intervention involved a single drug-gene interaction |
| DiFrancia 2019 (24) | Wrong patient population: patients with malignancy, HIV, HCV and HBV |
| Dorfman 2013 (25) | Wrong intervention: no intervention |
| Emmelhainz 2018 (26) | Conference abstract: full-text study unobtainable |
| Forester 2020 (17) | Wrong patient population: no evidence of multimorbidity or polypharmacy |
| Frank 2019 (27) | Wrong intervention: intervention lacked a pharmacogenetics component |
| Freeman 2017 (28) | Conference abstract: full-text study unobtainable |
| French 2010 (29) | Wrong intervention: intervention involved a single drug-gene interaction |
| Gibbs 2019 (30) | Duplicate: duplicate conference abstract for an unobtainable full-text study ((31)) |
| Greden 2018 (32) | Conference abstract: full-text study retrieved ((33)) |
| Greden 2019 (33) | Wrong patient population: no evidence of multimorbidity or polypharmacy |
| Greden 2019 (34) | Duplicate: duplicate conference abstract for a retrieved full-text study ((33)) |
| Greden 2019 (35) | Duplicate: duplicate conference abstract for a retrieved full-text study ((33)) |
| Guchelaar 2017 (36) | Conference abstract: full-text study unobtainable as study is ongoing ((37)) |
| Hall-Flavin 2012 (38) | Wrong patient population: no evidence of multimorbidity or polypharmacy |
| Hall-Flavin 2013 (39) | Wrong patient population: no evidence of multimorbidity or polypharmacy |
| Harada 2017 (40) | Wrong intervention: intervention involved a single drug-gene interaction |
| Herbert 2017 (41) | Duplicate: duplicate protocol for a retrieved full-text study ((42)) |
| Herbert 2018 (43) | Protocol: full-text study retrieved ((42)) |
| Holland 2019 (44) | Wrong intervention: intervention lacked a pharmacogenetics component |
| Jain 2017 (45) | Conference abstract: full-text study unobtainable |
| Jaspers 2019 (46) | Conference abstract: full-text study unobtainable |
| Ji 2016 (8) | Wrong patient population: no evidence of multimorbidity or polypharmacy |
| Keine 2018 (47) | Wrong intervention: intervention lacked a pharmacogenetics component |
| Kennedy 2018 (48) | Wrong intervention: no intervention |
| Kimmel 2013 (49) | Conference abstract: full-text study retrieved ((50)) |
| Kimmel 2013 (50) | Wrong intervention: intervention involved a single drug-gene interaction |
| Kosti 2018 (51) | Wrong intervention: no intervention |
| Lamont 2018 (52) | Wrong intervention: intervention lacked a pharmacogenetics component |
| Lee 2016 (53) | Conference abstract: full-text study retrieved ((54)) |
| Leroux 2018 (55) | Wrong intervention: intervention lacked a pharmacogenetics component |
| Mayhew 2017 (56) | Conference abstract: full-text study excluded during screening process ((57)) |
| NCT01184300 2010 (58) | Protocol: full-text study retrieved ((59)) |
| NCT01633957 2012 (60) | Protocol: full-text study retrieved ((61)) |
| NCT02428660 2015 (62) | Protocol: full-text study retrieved and included ((63)) |
| NCT03537547 2018 (64) | Protocol: full-text study unobtainable (trial terminated) |
| NCT03597165 2018 (65) | Protocol: full-text study retrieved ((66)) |
| Paul 2017 (67) | Conference abstract: full-text study unobtainable |
| Pérez 2016 (68) | Conference abstract: full-text study retrieved ((69)) |
| Pérez 2017 (69) | Wrong patient population: no evidence of multimorbidity or polypharmacy |
| Pink 2014 (2) | Wrong patient population: no evidence of multimorbidity or polypharmacy |
| Ray 2015 (70) | Conference abstract: full-text studies retrieved ((3, 71-73)) |
| Roberts 2012 (59) | Wrong intervention: intervention involved a single drug-gene interaction |
| Roe 2018 (74) | Conference abstract: full-text study unobtainable |
| Roth 2013 (75) | Wrong intervention: intervention lacked a pharmacogenetics component |
| Ruano 2020 (76) | Wrong patient population: no evidence of multimorbidity or polypharmacy |
| Shickh 2019 (66) | Wrong patient population: patients with malignancy |
| Sutherland 2017 (77) | Wrong intervention: intervention lacked a pharmacogenetics component |
| Sweet 2017 (78) | Wrong patient population: no evidence of multimorbidity or polypharmacy |
| Sylvia 2014 (79) | Wrong intervention: intervention lacked a pharmacogenetics component |
| Tanner 2018 (42) | Wrong patient population: no evidence of multimorbidity or polypharmacy |
| Tiwari 2019 (80) | Conference abstract: full-text studies retrieved ((42, 73)) |
| Tylee 2012 (81) | Protocol: full-text study retrieved ((6)) |
| vanBronswijk 2019 (82) | Wrong intervention: intervention lacked a pharmacogenetics component |
| VanDeventer 2017 (83) | Conference abstract: full-text study retrieved ((15)) |
| Vassy 2014 (84) | Wrong patient population: no evidence of multimorbidity or polypharmacy |
| Walker 2019 (85) | Conference abstract: full-text study unobtainable |
| Winkelmann 2001 (86) | Wrong patient population: no evidence of multimorbidity or polypharmacy |
| Winner 2013 (71) | Wrong patient population: no evidence of multimorbidity or polypharmacy |
| Winner 2013 (72) | Wrong patient population: no evidence of multimorbidity or polypharmacy |
| Winner 2015 (73) | Wrong patient population: no evidence of multimorbidity or polypharmacy |
| Wu 2019 (31) | Conference abstract: full-text study unobtainable |
| Xu 2018 (61) | Wrong patient population: no evidence of multimorbidity or polypharmacy |
| Zastrozhin 2018 (87) | Wrong patient population: no evidence of multimorbidity or polypharmacy |
| Zastrozhin 2019 (88) | Wrong intervention: intervention involved a single drug-gene interaction |
| Zastrozhin 2019 (89) | Conference abstract: full-text study retrieved ((88)) |
| Zastrozhin 2019 (90) | Duplicate: duplicate conference abstract for a retrieved full-text study ((88)) |
| Zastrozhin 2019 (91) | Duplicate: duplicate conference abstract for a retrieved full-text study ((88)) |
| Zintchouk 2016 (92) | Wrong intervention: intervention lacked a pharmacogenetics component |

# Supplementary Table 2: Risk of bias assessments

| ***Risk of bias – RoB 2*** | **Elliott *et al.* (93)** | |
| --- | --- | --- |
| **Bias** | **Authors’ judgement** | **Support for judgement** |
| Bias arising from the randomisation process | Low risk of bias | Randomly assigned to intervention and control groups; baseline differences did not suggest any issues; allocation sequence was random and concealed |
| Bias due to deviations from the intended intervention (assignment) | Low risk of bias | Open-label trial; no concern due to the nature of the study; the principles of an intention-to-treat analysis were followed |
| Bias due to missing outcome data | Some concerns | Outcome assessment appears to have been conducted for each participant; presence of missing data and a sensitivity analysis was not conducted |
| Bias in measurement of the outcome | High risk of bias | The method of measuring the outcome seems appropriate and was the same across two groups; outcome assessors were aware of the intervention received; post-hoc amendments to outcomes with inclusion and exclusion; death was incorporated as a component of the primary endpoint post-hoc |
| Bias in selection of the reported result | Some concerns | The prespecified analysis plan was amended post-hoc; unclear if the reported effect estimates were selected on the basis of results from multiple outcome measurements |
| Other bias | None to report | None to report |
| Overall risk of bias |  | High risk of bias - the trial is judged to be at high risk of bias in at least one domain for this result |

##

| ***Risk of bias – RoB 2*** | **Kim *et al.* (63)** | |
| --- | --- | --- |
| **Bias** | **Authors’ judgement** | **Support for judgement** |
| Bias arising from the randomisation process | Low risk of bias | Allocation sequence was random and concealed; participants assigned to groups based on birth year; marginally significant baseline difference showing greater number of ADRs in the PGx group than the other two arms (p = 0.06) |
| Bias due to deviations from the intended intervention (assignment) | Low risk of bias | Open-label trial; no concern due to the nature of the study; post-hoc analysis – high drop-out from the PGx arm resulted in reassignment of participants to control as the intention-to-treat analysis did not provide insight into the utility of the PGx service; deviations unlikely to have affected the outcome |
| Bias due to missing outcome data | Some concerns | Data appears to be available for all participants; protocol not publicly available; trial registry lists outcomes that are missing: number of ADRs, QoL, major event risk reduction; loss to follow-up not reported |
| Bias in measurement of the outcome | Low risk of bias | Unblinded pharmacists conducted initial medication review session; however, baseline differences were not statistically significant; Blinded pharmacists conducted secondary outcome (level of seriousness) measurement |
| Bias in selection of the reported result | Some concerns | Trial registry shows deviations from intended outcomes in the published work; however, it is a post-hoc analysis; unclear if the reported effect estimates were selected on the basis of results from multiple outcome measurements (different scales for severity ratings could be used) |
| Other bias |  | None to report |
| Overall risk of bias |  | Some concerns - the trial is judged to raise some concerns in at least one domain for this result, but not to be at high risk of bias for any domain |

| ***Risk of bias –***  ***ROBINS-I*** | **Brixner *et al.* (11)** | |
| --- | --- | --- |
| **Bias** | **Authors’ judgement** | **Support for judgement** |
| Bias due to confounding | Low risk of bias | Control group propensity score matched to intervention group by age, gender, D’Hoore-Charlson comorbidity index score for specific morbidities, and for high-risk CYP450 medications |
| Bias in selection of participants into the study | Low risk of bias | Participants selection satisfactory (patients were included based on prior-agreed inclusion criteria); control group patients matched to intervention group patients on key characteristics; start of follow-up and start of intervention coincides for most participants |
| Bias in classification of interventions | Low risk of bias | Intervention groups clearly defined, with a prospective intervention group and retrospective control group; classification of intervention status unlikely to have been affected by knowledge of the outcome |
| Bias due to deviations from the intended intervention | Low risk of bias | No deviations from the intended intervention (intervention as per trial registry); protocol not publicly available |
| Bias due to missing data | Low risk of bias | Data appears to be available for all participants; no information on loss to follow-up |
| Bias in measurement of outcomes | Moderate risk of bias | No evidence of blinding or outcome/participant exclusion; outcomes reported on as per trial registry; outcome assessments may have differed between groups, with different resources available for data collection in the intervention and control group |
| Bias in selection of the reported result | Low risk of bias | There is sufficient evidence that reported outcomes arise from intended outcomes, analyses and cohorts based on the PGx intervention |
| Other bias |  | None to report |
| Overall risk of bias |  | Moderate risk of bias - the study is sound for a non-randomised study but cannot be considered comparable to a well-performed randomised trial |

| ***Risk of bias –***  ***ROBINS-I*** | **van der Wouden *et al.* (94)** | |
| --- | --- | --- |
| **Bias** | **Authors’ judgement** | **Support for judgement** |
| Bias due to confounding | Moderate risk of bias | Potential for confounding (groups not controlled for patient factors); the impact of a PGx confounded when patients are on multiple medications and have multiple comorbidities (increased likelihood of finding a useful effect); there is no missing information on identified potential confounding variables. |
| Bias in selection of participants into the study | Moderate risk of bias | Participants selection satisfactory (patients were included based on prior-agreed inclusion criteria); follow-up and start of intervention may not coincide for most participants (follow-up duration is mentioned to be a mean of 2.5 years); adjustment techniques used to correct for presence of selection biases |
| Bias in classification of interventions | Low risk of bias | Intervention groups clearly defined; there are no deviations from intervention and knowledge of intervention will have no impact on outcome; only one intervention group with stratification into groups performed for comparisons |
| Bias due to deviations from the intended intervention | Low risk of bias | No deviations from intended intervention are reported |
| Bias due to missing data | Low risk of bias | Due to the cross-sectional nature of the study, no patients were lost to follow-up |
| Bias in measurement of outcomes | Moderate risk of bias | Outcome measure could be influenced by knowledge of intervention received; differences could not be concluded due to high healthcare professional adherence to guidelines |
| Bias in selection of the reported result | Low risk of bias | There is sufficient evidence that reported outcomes arise from intended outcomes, analyses and cohorts based on the PGx intervention |
| Other bias |  | None to report |
| Overall risk of bias |  | Moderate risk of bias - the study is sound for a non-randomised study but cannot be considered comparable to a well-performed randomised trial |

# Supplementary Table 3: Study search strategies

## 3.1 PubMed search strategy

| 1. “pharmacogenetics"[Text Word] 2. "pharmacogenetic variants"[Text Word] 3. "pharmacogenetic testing"[Text Word] 4. "genetic testing"[Text Word] 5. "precision medicine"[Text Word] 6. "personalised medicine"[Text Word] 7. "personalized medicine"[Text Word] 8. "pharmacogenomics"[Text Word] 9. "genetic variation"[Text Word] 10. "genetic polymorphism"[Text Word] 11. "clinical enzyme tests"[Text Word] 12. "hematological tests"[Text Word] 13. "haematological tests"[Text Word] 14. "buccal swabs"[Text Word] 15. "cheek swab"[Text Word] 16. or/1-15 17. multimorbid*[Text Word] 18. multi-morbid*[Text Word] 19. multi morbid*[Text Word] 20. "multiple chronic conditions"[Text Word] 21. "multiple chronic diseases"[Text Word] 22. "multiple chronic illnesses"[Text Word] 23. chronic condition*[Text Word] 24. chronic disease*[Text Word] 25. chronic illness*[Text Word] 26. "chronic care"[Text Word] | 1. chronic syndrome*[Text Word] 2. chronic disorder*[Text Word] 3. "multidisease"[Text Word] 4. multi-disease[Text Word] 5. multiple condition*[Text Word] 6. multiple disease*[Text Word] 7. multiple illness*[Text Word] 8. multiple syndrome*[Text Word] 9. multiple disorder*[Text Word] 10. comorbid*[Text Word] 11. co-morbid*[Text Word] 12. co morbid*[Text Word] 13. "polypharmacy"[Text Word] 14. "polymedication"[Text Word] 15. "polytherapy"[Text Word] 16. polypharma*[Text Word] 17. multiple medic*[Text Word] 18. chronic medic*[Text Word] 19. long term medic*[Text Word] 20. multiple drug*[Text Word] 21. concomitant medic*[Text Word] 22. concurrent medic*[Text Word]) 23. or/17-48 24. full text[sb] 25. 16 and 49 and 50 |
| --- | --- |

## 3.2 EMBASE search strategy

| 1. pharmacogenetics:ti,ab,kw 2. 'pharmacogenetic variant':ti,ab,kw 3. 'pharmacogenetic testing':ti,ab,kw 4. 'genetic screening':ti,ab,kw 5. 'precision medicine':ti,ab,kw 6. 'personalised medicine':ti,ab,kw 7. 'personalized medicine':ti,ab,kw 8. pharmacogenomics:ti,ab,kw 9. 'genetic variation':ti,ab,kw 10. 'genetic polymorphism':ti,ab,kw 11. 'clinical enzyme tests':ti,ab,kw 12. 'hematological tests':ti,ab,kw 13. 'haematological tests':ti,ab,kw 14. 'buccal swabs':ti,ab,kw 15. 'cheek swab':ti,ab,kw 16. or/1-15 17. multimorbid*:ti,ab,kw 18. 'multi morbid*':ti,ab,kw 19. 'multiple chronic conditions':ti,ab,kw 20. 'multiple chronic diseases':ti,ab,kw 21. 'multiple chronic illnesses':ti,ab,kw 22. 'chronic condition*':ti,ab,kw 23. 'chronic disease*':ti,ab,kw 24. 'chronic illness*':ti,ab,kw | 1. 'chronic care':ti,ab,kw 2. 'chronic syndrome*':ti,ab,kw 3. 'chronic disorder*':ti,ab,kw 4. multidisease:ti,ab,kw 5. 'multi disease':ti,ab,kw 6. 'multiple condition*':ti,ab,kw 7. 'multiple disease*':ti,ab,kw 8. 'multiple illness*':ti,ab,kw 9. 'multiple syndrome*':ti,ab,kw 10. 'multiple disorder*':ti,ab,kw 11. comorbid*:ti,ab,kw 12. 'co morbid*':ti,ab,kw 13. polypharmacy:ti,ab,kw 14. polymedication:ti,ab,kw 15. polytherapy:ti,ab,kw 16. polypharma*:ti,ab,kw 17. 'multiple medic*':ti,ab,kw 18. 'chronic medic*':ti,ab,kw 19. 'long term medic*':ti,ab,kw 20. 'multiple drug*':ti,ab,kw 21. 'concomitant medic*':ti,ab,kw 22. 'concurrent medic*':ti,ab,kw 23. or/17-46 24. 16 and 47 |
| --- | --- |

## 3.3 CENTRAL – Cochrane Central Register of Controlled Trials search strategy

| #1 MeSH descriptor: [Pharmacogenetics] explode all trees  #2 MeSH descriptor: [Pharmacogenomic Variants] explode all trees  #3 MeSH descriptor: [Pharmacogenomic Testing] explode all trees  #4 MeSH descriptor: [Genetic Testing] explode all trees  #5 MeSH descriptor: [Precision Medicine] explode all trees  #6 personalised medicine:ti,ab,kw  #7 personalized medicine:ti,ab,kw  #8 pharmacogenomics:ti,ab,kw  #9 MeSH descriptor: [Genetic Variation] explode all trees  #10 MeSH descriptor: [Polymorphism, Genetic] explode all trees  #11 MeSH descriptor: [Clinical Enzyme Tests] explode all trees  #12 MeSH descriptor: [Hematologic Tests] explode all trees  #13 haematological tests:ti,ab,kw  #14 buccal swabs:ti,ab,kw  #15 cheek swabs:ti,ab,kw  #16 #1 or #2 or #3 or #4 or #5 or #6 or #7 or #8 or #9 or #10 or #11 or #12 or #13 or #14 or #15  #17 multimorbid*:ti,ab,kw  #18 multi-morbid*:ti,ab,kw  #19 multi morbid*:ti,ab,kw  #20 MeSH descriptor: [Multiple Chronic Conditions] explode all trees  #21 multiple chronic diseases:ti,ab,kw  #22 multiple chronic illnesses:ti,ab,kw | #23 chronic condition*:ti,ab,kw  #24 chronic disease*:ti,ab,kw  #25 chronic illness*:ti,ab,kw  #26 chronic care:ti,ab,kw  #26 chronic syndrome*:ti,ab,kw  #28 chronic disorder*:ti,ab,kw  #29 multidisease:ti,ab,kw  #30 multi-disease:ti,ab,kw  #31 multiple condition*:ti,ab,kw  #32 multiple disease*:ti,ab,kw  #33 multiple illness*:ti,ab,kw  #34 multiple syndrome*:ti,ab,kw  #35 multiple disorder*:ti,ab,kw  #36 comorbid*:ti,ab,kw  #37 co-morbid*:ti,ab,kw  #38 co morbid*:ti,ab,kw  #39 MeSH descriptor: [Polypharmacy] explode all trees  #40 polymedication:ti,ab,kw  #41 polytherapy:ti,ab,kw  #42 polypharma*:ti,ab,kw  #43 multiple medic*:ti,ab,kw  #44 chronic medic*:ti,ab,kw  #45 long term medic*:ti,ab,kw  #46 multiple drug*:ti,ab,kw  #47 concomitant medic*:ti,ab,kw  #48 concurrent medic*:ti,ab,kw  #49 #17 or #18 or #19 or #20 or #21 or #22 or #23 or #24 or #25 or #26 or #27 or #28 or #29 or #30 or #31 or #32 or #33 or #34 or #35 or #36 or #37 or #38 or #39 or #40 or #41 or #42 or #43 or #44 or #45 or #46 or #47 or #48  #50 #16 and #49 |
| --- | --- |

## 3.4 CINAHL – Cumulative Index to Nursing and Allied Health Literature; AMED – The Allied and Complimentary Medicine Database; and PsycInfo search strategy

S1 TI ( pharmacogenetics OR pharmacogenetic variants OR pharmacogenetic testing OR genetic testing OR precision medicine OR personalised medicine OR personalized medicine OR pharmacogenomics OR genetic variation OR genetic polymorphism OR clinical enzyme tests OR hematological tests OR haematological tests OR buccal swabs OR cheek swab ) OR AB ( pharmacogenetics OR pharmacogenetic variants OR pharmacogenetic testing OR genetic testing OR precision medicine OR personalised medicine OR personalized medicine OR pharmacogenomics OR genetic variation OR genetic polymorphism OR clinical enzyme tests OR hematological tests OR haematological tests OR buccal swabs OR cheek swab )

S2 TI ( multimorbid* OR multi-morbid* OR multi morbid* OR multiple chronic conditions OR multiple chronic diseases OR multiple chronic illnesses OR chronic condition* OR chronic disease* OR chronic illness* OR chronic care OR chronic syndrome* OR chronic disorder* OR multidisease OR multi-disease OR multiple condition* OR multiple disease* OR multiple illness* OR multiple syndrome* OR multiple disorder* OR comorbid* OR co-morbid OR co morbid* OR polypharmacy OR polymedication OR polytherapy OR polypharma* OR multiple medic* OR chronic medic* OR long term medic* OR multiple drug* OR concomitant medic* OR concurrent medic* ) OR AB ( multimorbid* OR multi-morbid* OR multi morbid* OR multiple chronic conditions OR multiple chronic diseases OR multiple chronic illnesses OR chronic condition* OR chronic disease* OR chronic illness* OR chronic care OR chronic syndrome* OR chronic disorder* OR multidisease OR multi-disease OR multiple condition* OR multiple disease* OR multiple illness* OR multiple syndrome* OR multiple disorder* OR comorbid* OR co-morbid OR co morbid* OR polypharmacy OR polymedication OR polytherapy OR polypharma* OR multiple medic* OR chronic medic* OR long term medic* OR multiple drug* OR concomitant medic* OR concurrent medic*)

S3 S1 AND S2

**Limiters –** Full text; exclude MEDLINE records

**Expanders –** Apply related words

**Search modes –** Boolean/Phrase

REFERENCES

1. Al-Zubiedi S, Hanson A, Jorgensen A, Pirmohamed M. Impact of Pharmacogenetics on the Costs of Managing Adverse Events with Warfarin: A Prospective Analysis. Value Health. 2009;12(3):A3-A4.

2. Pink J, Pirmohamed M, Lane S, Hughes DA. Cost-Effectiveness of Pharmacogenetics-Guided Warfarin Therapy Vs. Alternative Anticoagulation in Atrial Fibrillation. Clin Pharmacol Ther. 2014;95(2):199-207.

3. Altar CA, Carhart J, Allen JD, Hall-Flavin DK, Dechairo BM, Winner JG. Clinical Validity: Combinatorial Pharmacogenomics Predicts Antidepressant Responses and Healthcare Utilizations Better than Single Gene Phenotypes. Pharmacogenomics J. 2015;15(5):443-51.

4. Anderson JL, Horne BD, Stevens SM, Grove AS, Barton S, Nicholas ZP, et al. Randomized Trial of Genotype-Guided Versus Standard Warfarin Dosing in Patients Initiating Oral Anticoagulation. Circulation. 2007;116(22):2563‐70.

5. Anderson A, Limdi M, Cavallari L, Baird M, Allon M, Beasley M, et al. Warfarin Dosing in Patients with Impaired Renal Function. Am J Kidney Dis. 2010;55(4):A75.

6. Barley EA, Walters P, Haddad M, Phillips R, Achilla E, McCrone P, et al. The UPBEAT Nurse-Delivered Personalized Care Intervention for People with Coronary Heart Disease who Report Current Chest Pain and Depression: A Randomised Controlled Pilot Study. PLoS One. 2014;9(6):e98704.

7. Bielinski SJ, Olson J, Pathak J, Weinshilboum RM, Wang L, Lyke KJ, et al. Preemptive Genotyping for Personalized Medicine: Design of the Right Drug, Right Dose, Right Time-Using Genomic Data to Individualize Treatment Protocol. Mayo Clin Proc. 2014;89(1):25-33.

8. Ji Y, Skierka JM, Blommel JH, Moore BE, VanCuyk DL, Bruflat JK, et al. Preemptive Pharmacogenomic Testing for Precision Medicine: A Comprehensive Analysis of Five Actionable Pharmacogenomic Genes Using Next-Generation DNA Sequencing and a Customized CYP2D6 Genotyping Cascade. The Journal of Molecular Diagnostics. 2016;18(3):438-45.

9. Bielinski SJ, Olson J, Pathak J, Weinshilboum RM, Wang L, Lyke KJ, et al. Preemptive Genotyping for Personalized Medicine: Design of the Right Drug, Right Dose, Right Timedusing Genomic Data to Individualize Treatment Protocol. Mayo Clin Proc. 2014;89(1):25-33.

10. Biskupiak J, Biltaji E, Bress A, Ye X, Unni S, Newman R, et al. Cost-Consequence Analysis for Pharmacogenetic Testing in an Elderly Population. J Manag Care Spec Pharm. 2015;21:S81.

11. Brixner D, Biltaji E, Bress A, Unni S, Ye X, Mamiya T, et al. The Effect of Pharmacogenetic Profiling with a Clinical Decision Support Tool on Healthcare Resource Utilization and Estimated Costs in the Elderly Exposed to Polypharmacy. J Med Econ. 2016;19(3):213-28.

12. Biskupiak JE, Biltaji E, Bress A, Unni S, Ye X, Yu B, et al. Value Assessment for Genetic Testing of Drug Variation in an Elderly Population. Value Health. 2015;18(7):A747.

13. Boels AM, Hart HE, Rutten GE, Vos RC. Personalised Treatment Targets in Type 2 Diabetes Patients: The Dutch Approach. Prim Care Diabetes. 2017;11(1):71-7.

14. Bradley P, Shiekh M, Mehra V, Vrbicky K, Layle S, Olson MC, et al. Improved Efficacy with Targeted Pharmacogenetic-Guided Treatment of Patients with Depression and Anxiety: A Randomized Clinical Trial Demonstrating Clinical Utility. J Psychiatr Res. 2018;96:100-7.

15. Brown LC, Lorenz RA, Li J, Dechairo BM. Economic Utility: Combinatorial Pharmacogenomics and Medication Cost Savings for Mental Health Care in a Primary Care Setting. Clin Ther. 2017;39(3):592-602.e1.

16. Brown L, Forester B, Parikh S, Weisenbach S, Ajilore O, Vahia I, et al. Combinatorial Pharmacogenetic Testing Improves Response and Remission for Patients Over 65 with Depression who have Failed One Medication Trial. Am J Geriatr Psychiatry. 2020;28(4):S151-S2.

17. Forester BP, Parikh S, Weisenbach S, Ajilore O, Vahia I, Rothschild AJ, et al. Combinatorial Pharmacogenomic Testing Improves Outcomes for Older Adults With Depression. Am J Geriatr Psychiatry. 2020.

18. Burnette R, Simmons L, Snyderman R. Personalized Health Care as a Pathway for the Adoption of Genomic Medicine. J Pers Med. 2012;2(4):232-40.

19. Cervantes R, Lapp R. Optimal Blood Pressure Control is Achievable with Precision Medicine Delivered by the Serial Hypertension Clinic Approach-Improving Patient Adherence. J Hypertens. 2019;37:e74.

20. Charland SL, Agatep BC, Epstein R, Frueh FW, Herrera V, Devlin J, et al. Patient Knowledge of Pharmacogenetic Information Improves Adherence to Statin Therapy: Results of the Additional KIF6 Risk Offers Better Adherence to Statins (AKROBATS) Trial. J Am Coll Cardiol. 2012;59(13):E1848.

21. Charland SL, Agatep BC, Herrera V, Schrader B, Frueh FW, Ryvkin M, Shabbeer J, Devlin JJ, Superko HR, Stanek EJ. Providing Patients with Pharmacogenetic Test Results Affects Adherence to Statin Therapy: Results of the Additional KIF6 Risk Offers Better Adherence to Statins (AKROBATS) Trial. Pharmacogenomics J. 2014;14(3):272-80.

22. Chenoweth L, Forbes I, Fleming R, King MT, Stein-Parbury J, Luscombe G, et al. PerCEN: A Cluster Randomized Controlled Trial of Person-Centered Residential Care and Environment for People with Dementia. Int Psychogeriatr. 2014;26(7):1147‐60.

23. Clarke JA, Cutler M, Gong I, Schwarz UI, Freeman D, Dasgupta M. Cytochrome P450 2D6 Phenotyping in an Elderly Population with Dementia and Response to Galantamine in Dementia: A Pilot Study. Am J Geriatr Pharmacother. 2011;9(4):224-33.

24. Di Francia R, de Lucia V, Giordano A, Benincasa G, Mignano M, Berretta M. Pharmacogenomics as a tool to prevent drug-related hospitalization of elderly cardiology-oncology patients receiving chemotherapeutic agents and multiple symptomatic treatments: A pilot study planned for the Italian health system. Eur Rev Med Pharmacol Sci. 2019;23(19):8695-701.

25. Dorfman R, Khayat Z, Sieminowski T, Golden B, Lyons R. Application of personalized medicine to chronic disease: a feasibility assessment. Clin Transl Med. 2013;2(1):16.

26. Emmelhainz J, Adams K, Postolski J, Willoughby M. Outcomes of pharmacogenetic-directed antipsychotic medication therapy in older adults in long-term care population. Consult Pharm. 2018;33(10):585.

27. Frank F, Bjerregaard F, Bengel J, Bitzer EM, Heimbach B, Kaier K, et al. Local, Collaborative, Stepped and Personalised Care Management for Older People with Chronic Diseases (LoChro): Study Protocol of a Randomised Comparative Effectiveness Trial. BMC Geriatr. 2019;19(1):64.

28. Freeman K, Cohen C, Reinhardt M, Mani A, Ghoneim D. Pharmacogenetics-Guided Treatment of Mental Illness in Late-Life: A Case Series. Am J Geriatr Psychiatry. 2017;25(3):S144-S5.

29. French B, Joo J, Geller NL,Kimmel SE, Rosenberg Y, Anderson JL, et al. Statistical Design of Personalized Medicine Interventions: The Clarification of Optimal Anticoagulation through Genetics (COAG) Trial. Trials. 2010;11:108.

30. Gibbs M, Devery S, Wu K. Pharmacogenomics as an Elements of Precision Healthcare: The St Vincent's Experience. Twin Res Hum Genet. 2019;22(5):398.

31. Wu K, Gibbs M, Devery S. Pharmacogenomics as an Element of Precision Healthcare: The St Vincent's Experience. Twin Res Hum Genet. 2019;22(5):360.

32. Greden JF, Rothschild AJ, Zandy S, Thase M, Dunlop BW, DeBattista C, et al. Combinatorial Pharmacogenomics Significantly Improves Response and Remission for Major Depressive Disorder: A Double-Blind, Randomized Control Trial. Consult Pharm. 2018;33(10):599‐.

33. Greden JF, Parikh S, Rothschild AJ, Thase ME, Dunlop BW, DeBattista C, et al. Impact of Pharmacogenomics on Clinical Outcomes in Major Depressive Disorder in the GUIDED Trial: A Large, Patient- and Rater-Blinded, Randomized, Controlled Study. J Psychiatr Res. 2019;111:59-67.

34. Greden JF, Rothschild AJ, Thase M, Dunlop BW, DeBattista C, Conway CR, et al. Combinatorial Pharmacogenomics to Guide Treatment Selection for Major Depressive Disorder: A Large, Blinded, Randomized Controlled Trial. CNS Spectr. 2019;24(1):202‐3.

35. Greden JF, Rothschild AJ, Thase M, Dunlop BW, DeBattista C, Conway CR, et al. 49 Combinatorial Pharmacogenomics to Guide Treatment Selection for Major Depressive Disorder: a Large, Blinded, Randomized Controlled Trial. CNS Spectr. 2019;24(1):202‐3.

36. Guchelaar H. Pre-Emptive Genotyping for Preventing ADRs. Drug Saf. 2017;40(10):1036‐7.

37. van der Wouden CH, Cambon-Thomsen A, Cecchin E, Cheung KC, Dávila-Fajardo CL, Deneer VH, et al. Implementing Pharmacogenomics in Europe: Design and Implementation Strategy of the Ubiquitous Pharmacogenomics Consortium. Clin Pharmacol Ther. 2017;101(3):341-58.

38. Hall-Flavin DK, Winner JG, Allen JD, Jordan JJ, Nesheim RS, Synder KA, et al. Using a Pharmacogenomic Algorithm to Guide the Treatment of Depression. Transl Psychiatry. 2012;2(10):e172-e.

39. Hall-Flavin DK, Winner JG, Allen JD, Carhart JM, Proctor B, Synder KA, et al. Utility of Integrated Pharmacogenomic Testing to Support the Treatment of Major Depressive Disorder in a Psychiatric Outpatient Setting. Pharmacogenet Genomics. 2013;23(10):535-48.

40. Harada S, Zhou Y, Duncan S, Armstead AR, Coshatt GM, Dillon C, et al. Precision Medicine at the University of Alabama at Birmingham: Laying the Foundational Processes Through Implementation of Genotype-Guided Antiplatelet Therapy. Clin Pharmacol Ther. 2017;102(3):493-501.

41. Herbert D, Neves-Pereira M, Baidya R, Cheema S, Groleau S, Shahmirian A, et al. Genetic Testing as a Supporting Tool in Prescribing Psychiatric Medication: Design and Protocol of the IMPACT Study. J Psychiatr Res. 2017.

42. Tanner JA, Davies P, Voudouris NC, Shahmirian A, Herbert D, Braganza N, et al. Combinatorial Pharmacogenomics and Improved Patient Outcomes in Depression: Treatment by Primary Care Physicians or Psychiatrists. J Psychiatr Res. 2018;104:157-62.

43. Herbert D, Neves-Pereira M, Baidya R, Cheema S, Groleau S, Shahmirian A, et al. Genetic Testing as a Supporting Tool in Prescribing Psychiatric Medication: Design and Protocol of the IMPACT Study. J Psychiatr Res. 2018;96:265-72.

44. Holland AE, Lee A. Precision Medicine, Healthy Living and the Complex Patient: Managing the Patient With Multimorbidity. Prog Cardiovasc Dis. 2019;62(1):29-33.

45. Jain S, Danesh A, Laroche M, Bakhtiari S, Maxwell C, Al-Khalili Y, et al. Using Pharmacogenomics as an Effective Tool in Minimizing Drug Related Side Effects in Patients with Chronic Regional Pain Syndrome: A Feasibility Study. J Pain. 2017;18(4):S58-S9.

46. Jaspers NEM, Visseren FLJ, Van Der Graaf Y, Damman OC, Smulders YM, Dorresteijn JAN. Effects of Personalized Therapy-Effect Predictions on Statin Treatment Decisions by Patients and Physicians: A Three-Armed, Blinded, Randomized Controlled Trial. Eur Heart J. 2019;40:255‐.

47. Keine D, Walker JQ, Kennedy BK, Sabbagh MN. Development, Application, and Results from a Precision-medicine Platform that Personalizes Multi-modal Treatment Plans for Mild Alzheimer's Disease and At-risk Individuals. Curr Aging Sci. 2018;11(3):173-81.

48. Kennedy GJ. Added Value of the Personalized Intervention for Depressed Patients with COPD. Am J Geriatr Psychiatry. 2018;26(2):172-3.

49. Kimmel SE, French B, Kasner S, Johnson JA, Anderson JL, Gage BF, et al. The Clarification of Optimal Anticoagulation Through Genetics (COAG) Trial. Circulation. 2013;128(24):2711.

50. Kimmel SE, French B, Kasner SE, Johnson JA, Anderson JL, Gage BF, et al. A Pharmacogenetic Versus a Clinical Algorithm for Warfarin Dosing. N Engl J Med. 2013;369(24):2283-93.

51. Kosti I, Sirota M. Electronic Medical Records Enable Precision Medicine Approaches for Celiac Disease. J Pediatr Gastroenterol Nutr. 2018;67(4):434-5.

52. Lamont A, Lyons MD, Jaki T, Stuart E, Feaster DJ, Tharmaratnam K, et al. Identification of Predicted Individual Treatment Effects in Randomized Clinical Trials. Stat Methods Med Res. 2018;27(1):142‐57.

53. Lee Y, Danahey K, Ratain MJ, Meltzer DO, O'Donnell PH. Analysis of Clinically Actionable Preemptive Pharmacogenomic (PGX) Information to Impact in Hospital Prescribing. Clin Pharmacol Ther. 2016;99:S107.

54. Lee YM, Danahey K, Knoebel RW, Ratain MJ, Meltzer DO, O'Donnell PH. Analysis of Comprehensive Pharmacogenomic Profiling to Impact In-Hospital Prescribing. Pharmacogenet Genomics. 2019;29(2):23-30.

55. Leroux E, Beaudet L, Boudreau G, Eghtesadi M, Marchand L, Pim H, Chagnon M. A Nursing Intervention Increases Quality of Life and Self-Efficacy in Migraine: a 1-Year Prospective Controlled Trial. Headache. 2018;58(2):260‐74.

56. Mayhew M, Jablonski M, Li J, Dechairo B, Healthson A. Combinatorial Pharmacogenomics Reduces Polypharmacy and Medication Cost in Elderly Patients with Anxiety and Depression. Am J Geriatr Psychiatry. 2017;25(3):S143-S4.

57. Jablonski MR, Lorenz R, Li J, Dechairo BM. Economic Outcomes Following Combinatorial Pharmacogenomic Testing for Elderly Psychiatric Patients. J Geriatr Psychiatry Neurol. 2019:891988719892341.

58. NCT01184300. Re-assessment of anti-platelet therapy using an individualized strategy based on genetic evaluation. 2010. Available at: <https://clinicaltrials.gov/ct2/show/NCT01184300>. Accessed on: April 28, 2021.

59. Roberts JD, Wells GA, Le May MR, Labinaz M, Glover C, Froeschl M, et al. . Point-of-Care Genetic Testing for Personalisation of Antiplatelet Treatment (RAPID GENE): A Prospective, Randomised, Proof-of-Concept Trial. Lancet. 2012;379(9827):1705-11.

60. NCT01633957. A trial of genotype-based warfarin initiation in patients with mechanical prosthetic heart valve. 2012. Available at: <https://clinicaltrials.gov/ct2/show/NCT01633957>. Accessed on: April 28, 2021.

61. Xu Z, Zhang S, Huang M, Hu R, Li JL, Cen HJ, et al. Genotype-Guided Warfarin Dosing in Patients With Mechanical Valves: A Randomized Controlled Trial. Ann Thorac Surg. 2018;106(6):1774-81.

62. NCT02428660. Drug & gene interaction risk analysis with & without genetic testing among patients undergoing MTM. 2015. Available at: <https://clinicaltrials.gov/ct2/show/NCT02428660>. Accessed on: April 28, 2021.

63. Kim K, Magness J, Nelson R, Baron V, Brixner DI. Clinical Utility of Pharmacogenetic Testing and a Clinical Decision Support Tool to Enhance the Identification of Drug Therapy Problems Through Medication Therapy Management in Polypharmacy Patients. J Manag Care Spec Pharm. 2018;24(12):1250-9.

64. NCT03537547. Combinatorial pharmacogenomics testing in treatment-naïve major depressive disorder. 2018. Available at: <https://clinicaltrials.gov/ct2/show/NCT03537547>. Accessed on: April 28, 2021.

65. NCT03597165. Incidental genomics. 2018. Available at: <https://clinicaltrials.gov/ct2/show/NCT03597165>. Accessed on: April 28, 2021.

66. Shickh S, Clausen M, Mighton C, Salazar MG, Zakoor KR, Kodida R, et al. Health Outcomes, Utility and Costs of Returning Incidental Results from Genomic Sequencing in a Canadian Cancer Population: Protocol for a Mixed-Methods Randomised Controlled Trial. BMJ Open. 2019;9(10):e031092.

67. Paul S, Lu C, Block L. Personalized Pharmacotherapy in a Patient with Cytochrome P450 2D6 Polymorphism. J Gen Intern Med. 2017;32(2):S561-S2.

68. Pérez V, Espadaler J, Tuson M, Salavert A, Saiz J, Bobes J, et al. Effectiveness of Pharmacogenetic Information in the Treatment of Major Depressive Disorder: Results from the AB-GEN Randomized Clinical Trial. Eur Neuropsychopharmacol. 2016;26:S404-S5.

69. Pérez V, Salavert A, Espadaler J, Tuson M, Saiz-Ruiz J, Saez-Navarro C, et al. Efficacy of Prospective Pharmacogenetic Testing in the Treatment of Major Depressive Disorder: Results of a Randomized, Double-Blind Clinical Trial. BMC Psychiatry. 2017;17(1):250.

70. Ray B, Winner JG, Allen J, Carhart J, Dechairo B, Altar CA. Combinatorial Pharmacogenomics for Personalized Antidepressant Therapy: Clinical and Economic Validity and Utility. Neuropsychopharmacology. 2015;40:S497-S8.

71. Winner J, Allen JD, Altar CA, Spahic-Mihajlovic A. Psychiatric Pharmacogenomics Predicts Health Resource U tilization of Outpatients with Anxiety and Depression. Transl Psychiatry. 2013;3(3):e242.

72. Winner JG, Carhart J, Altar CA, Allen JD, Dechairo BM. A Prospective, Randomized, Double-Blind Study Assessing the Clinical Impact of Integrated Pharmacogenomic Testing for Major Depressive Disorder. Discov Med. 2013;16(89):219-27.

73. Winner JG, Carhart J, Altar CA, Goldfarb S, Allen JD, Lavezzari G, et al. Combinatorial Pharmacogenomic Guidance for Psychiatric Medications Reduces Overall Pharmacy Costs in a 1 Year Prospective Evaluation. Curr Med Res Opin. 2015;31(9):1633-43.

74. Roe N, Passariello C, Brown L, Li J, Jablonski M, Dechairo BM. Prospective Evaluation of the Economic Utility of Combinatorial Pharmacogenomics in Generalized Anxiety Disorder and Major Depressive Disorder. CNS Spectr. 2018;23(1):99-100.

75. Roth MT, Ivey J, Esserman DA, Crisp G, Kurz J, Weinberger M. Individualized Medication Assessment and Planning: Optimizing Medication U se in Older Adults in the Primary Care Setting. Pharmacotherapy. 2013;33(8):787-97.

76. Ruano G, Robinson S, Holford T, Mehendru R, Baker S, Tortora J et al. Results of the CYP-GUIDES Randomized Controlled Trial: Total Cohort and Primary Endpoints. Contemp Clin Trials. 2020;89.

77. Sutherland JJ, Morrison R, Daniels JS, Milne SB, Ryan TP. Managing Psychotropic Medications in Complex, Real-World Patients Using Comprehensive Therapeutic Drug Monitoring. ACS Chem Neurosci. 2017;8(8):1641-4.

78. Sweet K, Sturm A, Schmidlen T, McElroy J, Scheinfeldt L, Manickam K, et al. Outcomes of a Randomized Controlled Trial of Genomic Counseling for Patients Receiving Personalized and Actionable Complex Disease Reports. J Genet Couns. 2017;26(5):980-98.

79. Sylvia LG, Rabideau D, Nierenberg AA, Bowden CL, Friedman ES, Iosifescu DV, et al. The Effect of Personalized Guideline-Concordant Treatment on Quality of Life and Functional I mpairment in Bipolar Disorder. J Affect Disord. 2014;169:144‐8.

80. Tiwari A, Zai C, Zai G, Cheema S, Braganza N, Mueller D, et al. Combinatorial Pharmacogenomic Testing Improves Generalized Anxiety Disorder Treatment Response and Decreases Benzodiazapine Use. Eur Neuropsychopharmacol. 2019;29:S933.

81. Tylee A, Haddad M, Barley E, Ashworth M, Brown J, Chambers J, et al. A Pilot Randomised Controlled Trial of Personalised Care for Depressed Patients with Symptomatic Coronary Heart Disease in South London General Practices: The UPBEAT-UK RCT Protocol and Recruitment. BMC Psychiatry. 2012;12(1).

82. van Bronswijk SC, DeRubeis RJ, Lemmens L, Peeters FPML, Keefe JR, Cohen ZD, et al. Precision Medicine for Long-Term Depression Outcomes Using the Personalized Advantage Index Approach: Cognitive Therapy or Interpersonal Psychotherapy? Psychol Med. 2019:1‐11.

83. Van Deventer L, Brown L, Lorenz R, Li J, Dechairo B. Economic Utility: Combinatorial Pharmacogenomics and Medication Cost Savings for Mental Health Care in a Primary Care Setting. J Manag Care Spec Pharm. 2017;23:S56.

84. Vassy JL, Lautenbach DM, McLaughlin HM, Won Kong S, Christensen KD, Krier J, et al. The MedSeq Project: A Randomized Trial of Integrating Whole Genome Sequencing into Clinical Medicine. Trials. 2014;15:85.

85. Walker JQ, Zelek MC, Sabbagh MN. Addressing Polypharmacy Issues in an Elderly Population with Cognitive Impairment Using a Precision-Medicine Platform. Alzheimers Dement. 2019;15(7):P219.

86. Winkelmann BR, Marz W, Boehm BO, Zotz R, Hager J, Hellstern P, et al. Rationale and Design of the LURIC Study - A Resource for Functional Genomics, Pharmacogenomics and Long-Term Prognosis of Cardiovascular Disease. Pharmacogenomics. 2001;2(1 Suppl 1):S1-73.

87. Zastrozhin MS, Sorokin A, Agibalova TV, Grishina EA, Antonenko AP, Rozochkin IN, et al. Using a Personalized Clinical Decision Support System for Bromdihydrochlorphenylbenzodiazepine Dosing in Patients with Anxiety Disorders Based on the Pharmacogenomic Markers. Hum Psychopharmacol. 2018;33(6):e2677.

88. Zastrozhin M, Skyrabin V, Sorokin A, Buzik O, Bedina I, Grishina E, et al. Using a Pharmacogenetic Clinical Decision Support System to Improve Psychopharmacotherapy Dosing in Patients with Affective Disorders. Drug Metab Pers Ther. 2020.

89. Zastrozhin MS, Sorokin A, Grishina EA, Bryun EA, Sychev DA. Implementation of Clinical Decision Support System for Dosing in Psychopharmacotherapy in Patients with Affective Disorders Based on the Pharmacogenomic Markers. Int J Rheum Dis. 2019;22:38-9.

90. Zastrozhin M, Sychev D, Bryun E. Using a Personalized Clinical Decision Support System for Dosing in Psychopharmacotherapy in Patients with Affective Disorders Based on the Pharmacogenomic Markers. Eur Neuropsychopharmacol. 2019;29:S208-S9.

91. Zastrozhin M, Sychev D, Sorokin A, Ryzhikova K, Bryun E. Development and Testing of a Clinical Decision Support System for Dosing in Psychopharmacotherapy in Patients with Affective Disorders Based on the Pharmacogenomic Markers. Eur J Clin Pharmacol. 2019;75:S82.

92. Zintchouk D, Lauritzen T, Damsgaard EM. Comprehensive Geriatric Care in Elderly Referred to a Rehabilitation Unit - A Randomized Trial. Eur Geriatr Med. 2016;7:S104‐.

93. Elliott LS, Henderson J, Neradilek MB, Moyer NA, Ashcraft KC, Thirumaran RK. Clinical Impact of Pharmacogenetic Profiling with a Clinical Decision Support Tool in Polypharmacy Home Health Patients: A Prospective Pilot Randomized Controlled Trial. PLoS One. 2017;12(2):e0170905.

94. van der Wouden CH, Bank PCD, Özokcu K, Swen JJ, Guchelaar HJ. Pharmacist-Initiated Pre-Emptive Pharmacogenetic Panel Testing with Clinical Decision Support in Primary Care: Record of PGx Results and Real-World Impact. Genes. 2019;10(6):416.
